# Abdominal Wall Evisceration Coupled With Iliac Vascular Injury After Blunt Trauma

**DOI:** 10.7759/cureus.34917

**Published:** 2023-02-13

**Authors:** Joseph C Novack, Eric L Whitton, Randi N Smith, Jason D Sciarretta, Jonathan Nguyen

**Affiliations:** 1 Department of Medicine, Emory University School of Medicine, Atlanta, USA; 2 Department of Anesthesiology, Emory University School of Medicine, Atlanta, USA; 3 Department of Surgery, Emory University School of Medicine, Atlanta, USA; 4 Department of Surgery, Morehouse School of Medicine, Atlanta, USA

**Keywords:** blunt abdominal trauma, concomitant vascular injury, major vascular injury, abdominal evisceration, blunt force trauma

## Abstract

Abdominal evisceration after blunt trauma is uncommon and rarely survivable when coupled with a concomitant iliac vascular injury. Blunt abdominal injury is rarely a cause of abdominal evisceration but may, on occasion, present in patients affected by a unique or high-energy traumatic injury. In these instances, major vascular injury is exceedingly rare but is associated with a high mortality rate. Damage to important vessels that may present more subtly, such as iliac arterial injury, can still be lethal and are important to evaluate in the trauma workup for blunt evisceration. We report the case of a 20-year-old woman who survived an abdominal wall and vascular injury in a motor vehicle accident. Management of this unusual association is discussed.

## Introduction

Traumatic injuries are the most common causes of mortality afflicting the young population, with blunt trauma from car accidents among the most common method of injury [1]. Abdominal evisceration from blunt trauma is an exceedingly rare occurrence. Only a small percentage of patients affected by blunt trauma sustain injuries to their abdominal wall, and even fewer suffer the forces necessary to cause a total evisceration of the abdominal contents within [2]. Surgeons evaluating a patient with blunt abdominal injury may not immediately consider major vascular injury as it is exceedingly rare. Among cases of blunt abdominal trauma, it has been shown to occur only 3% of the time with injury to the iliac arteries rarely if ever recorded [3]. While prompt surgical intervention can save a patient with blunt abdominal evisceration, a missed concurrent major vascular injury can result in death. Iliac artery trauma specifically is associated with a mortality rate as high as 60%, and while shown to be exceedingly rare in the context of blunt trauma, it should be kept in mind during assessment [4]. Although management and outcomes may vary greatly when abdominal evisceration and major vascular injury occur simultaneously in one patient, expeditious identification of such injuries is critical to patient care. We discuss the management of a patient with concomitant abdominal wall evisceration and major vascular injury. This paper was presented as a poster at the 86th Annual Meeting of the Southeastern Surgical Congress on February 12, 2018.

## Case presentation

**Figure 1:**
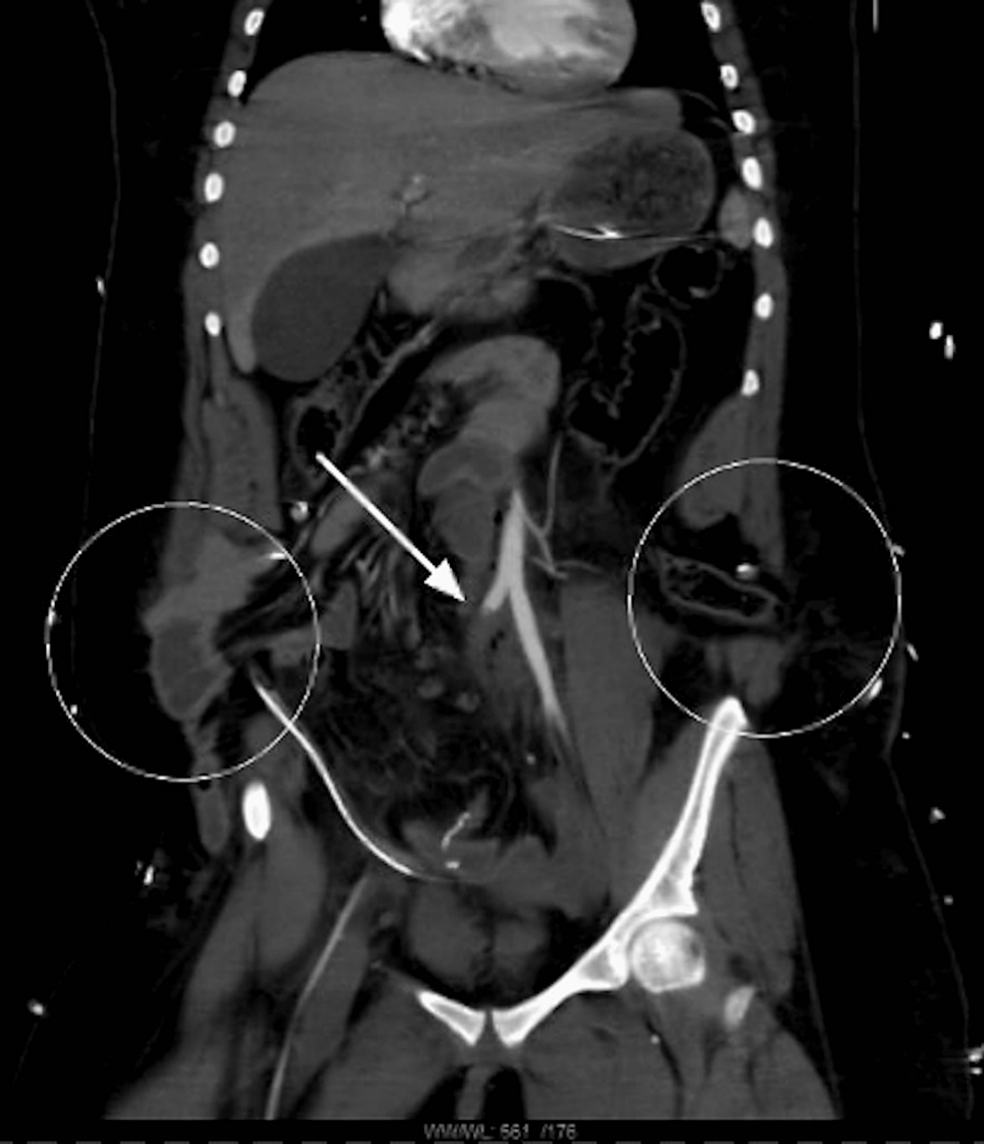
CT scan of the patient’s postoperative abdomen depicting iliac artery injury (arrow), with circles highlighting the ostomy and a hernia left behind. CT, computed tomography

A 20-year-old woman was the restrained passenger in the front seat during a motor vehicle collision. Upon arrival, she was tachycardic with a heart rate of 130 beats per minute, her primary survey was intact, and her Glasgow Coma Scale (GCS) was 15. On further evaluation, her entire abdominal wall was disrupted transversely, violating the peritoneum and resulting in an evisceration of all her small bowels. Abrasions on the remaining skin appeared consistent with a seatbelt sign across the lower abdomen. The patient was taken emergently to the operating room. In addition to the eviscerated bowel, there was already a significant loss of domain in the anterior abdominal wall. A degloving injury similar to a Morel Lavallee lesion that extended laterally to the retroperitoneum near the iliac crests bilaterally was identified at the level of the lap belt rests. On further exploration, the patient had a partially avulsed appendix and a completely devascularized segment of the proximal ileum and sigmoid colon in a traditional *bucket-handle* fashion. An appendectomy, small bowel resection, and sigmoidectomy were performed. Although the operative site had significant devascularized bowel and necrotic fat, there were no signs of feculent peritonitis or purulence. A Hartmann’s colostomy was matured superior to the transverse abdominal wall disruption. Two 19-French Blake drains were left in place, and the vertical midline fascia was reapproximated as best as possible to prevent further loss of domain. The skin was loosely reapproximated with staples. An immediate postoperative completion CT scan was performed to identify any additional injuries. It revealed complete occlusion of the right common iliac artery and no further significant injuries, as shown in Figure 1. The patient was subsequently taken back to the operating room for a right common iliac artery repair with an interposition polytetrafluoroethylene (PTFE) graft and complex abdominal wall reconstruction with biological mesh to reinforce the fascia. After the case, there were bounding peripheral pulses in the right lower extremity, and the compartments were soft.

In the intensive care unit, she was monitored for reperfusion injury and possible signs of compartment syndrome. Her only other complication was a persistent superficial wound infection, and the patient was subsequently discharged from the hospital to rehab at two weeks. At her three-month follow-up, her wounds had all healed and had no further untoward complications.

## Discussion

Abdominal evisceration due to penetrating injury is fairly common, with one case study reporting 66 cases of evisceration due to abdominal stab wounds alone in three years [5]. In cases of penetrating abdominal trauma, mortality is greatest in those who are affected by concomitant vascular injury [6]. However, complete evisceration of the abdominal contents in the setting of blunt trauma is exceedingly rare. The grading system for these cases is ranked from I to VI, I being subcutaneous tissue contusion and VI being complete dissociation of the abdominal wall and herniation of the contents within [7]. In recorded events with blunt trauma to the abdomen, less than 10% result in abdominal wall injury and less than 2% present with any amount of herniation. Fewer than 0.2% of patients sustain a grade VI injury with total evisceration [7,8]. These injuries can often result from the impact of a motor vehicle collision, causing an acute increase in abdominal pressure that culminates in the tearing of fascia and expulsion of contents [7-9]. In most described case studies and retrospective evaluations, an immediate CT scan followed by emergent laparotomy is recommended [7,10].

In a literature review of articles discussing abdominal evisceration due to blunt trauma, it is first apparent how unusual evisceration of the abdominal contents is due to a blunt impact. In one report of over 120,000 trauma cases in five years, only three cases of evisceration were found after blunt injury [8]. Only nine previous case studies were noted featuring abdominal evisceration due to blunt trauma, and among these, not a single case of concomitant evisceration and major vascular injury was discussed as in our findings [7,8,11-13]. One study described a small injury to the jejunal arteries via a tear in the mesentery following perineal small bowel evisceration secondary to blunt abdominal trauma, but it was not classified as a major vascular injury [14]. Similarly, aside from the injury to the aorta, blunt vascular injuries of the abdomen are exceedingly uncommon [15,16]. In our situation, we elected to use PTFE to help avoid a size mismatch and narrowing of the reconstruction. Despite the contaminated nature of the wound, synthetic grafts have demonstrated similarly low complication rates when compared to vein grafts [17].

We suspect that our patient’s injury was caused by her lap belt restraint. On impact, the force of the collision was distributed across her body through the straps of the shoulder and lap seatbelt, resulting in abdominal rupture and bowel evisceration. Seatbelts in blunt trauma are known to cause pancreatic injuries, duodenal injuries, and thoracic spine injuries, and in this setting, they may have caused abdominal evisceration [18].

In unusual cases with blunt abdominal evisceration, the major vascular injury should be excluded, and we suggest the following management. First, prompt operative management is required to ensure that there are no life-threatening injuries from hemorrhage and that any bowel spillage is contained. Second, an immediate CT scan should be conducted to identify and categorize all other injuries. Finally, the abdominal wall defect should be closed along with definitive management of all other injuries on time to prevent further retraction of the fascia. To preserve the abdominal wall and the patient’s bowel function, rapid closure of the abdomen may be necessary, and the use of a biologic mesh was indicated in this case to relieve tension.

## Conclusions

To our knowledge, there are no reported cases of patients who are affected by simultaneous abdominal wall evisceration and major vascular injury. Both abdominal evisceration and abdominal vascular injury are much more likely as a result of penetrating injury, but it is possible to encounter the two together due to blunt force injury. A very important factor in survival is surgical control of major hemorrhage, and due to the rare nature of arterial injury in blunt trauma, it is possible that a damaged artery could be initially overlooked in the operating room.
